# 68 Degrees: New York City’s Residential Heat and Hot Water Code as an Invisible Energy Policy

**DOI:** 10.1086/726711

**Published:** 2023-10-01

**Authors:** Rebecca Katherine Wright

**Affiliations:** Northumbria University

## Abstract

For over a century, New York’s Residential Heat and Hot Water Code has controlled the distribution of heat in New York City. Established in 1918 by New York’s Department of Health, it mandated that all residential and office spaces in the city be heated to sixty-eight degrees Fahrenheit at all times. Changes to it in the ensuing years sought not only to protect New Yorkers’ health but reflected pressures in New York’s fuel economy, which experienced periods of shortages and a transition from anthracite coal to oil that started between the two World Wars. Consequently, the standardization of sixty-eight degrees Fahrenheit reflected shifting assumptions about health and the “right to heat” for different communities over time, and the practical need to ensure affordable fuel for the city’s population. The Heat Code, accordingly, played a crucial role in shaping energy consumption in New York and helping to formulate an “invisible energy policy”—that is, a policy developed in non-energy fields, such as health and housing, that alters energy usage in important but inconspicuous ways, with important consequences for the environment and for social justice.

In October 1918, New York’s Department of Health added a new amendment to its Sanitary Code. All centrally-heated apartments and office spaces within the city had to be maintained at a minimum of sixty-eight degrees Fahrenheit, with an adequate supply of hot water.^1^ This ordinance remains as part of New York City law, as the city’s Residential Heat and Hot Water Code (hereafter referred to as the Heat Code).^2^ The Heat Code preserved the sixty-eight degrees minimum for tenanted residencies during the heating season (from October 1 to May 31). Over its hundred-year history, however, the Heat Code has gone through many modifications (dropping to sixty-five degrees between 1942 and 1956, for instance), and its seasonal parameters and temporal rhythm have been contested and altered several times. Despite the code’s origins as a public health measure, adjustments to the code have not always been driven by the goal of preserving the health of New Yorkers. Rather, they have reflected broader pressures in New York’s fuel economy, which experienced periods of shortages and a transition from anthracite coal to oil that started between the two World Wars. Over this period, the Heat Code has been used to balance bodily comfort with the limits of the physical environment and the pressures of the fuel economy.

Even though the Heat Code shaped New York’s thermal interior for over a century, we know little about its history, or its role in shaping New York’s mineral fuel network.^3^ This is surprising given that residential heat is a major part of New York’s energy consumption. Nearly three quarters of greenhouse gas emissions (GHG) emitted from residential homes in New York in 2016 came from heat and hot water alone.^4^ Part of the reason why the Heat Code has been overlooked is because, until recently, health policy has been neglected as a major factor in shaping energy demand. As Larissa Nicholls and Yolande Strengers have noted, health and energy have often been treated in fragmented policy fields with little dialogue between the two.^5^ This separation exists not only at a policy level, but also in academic discussions; energy historians have yet to fully examine the impact of health policies on the development of carbon networks. Scholars have traced the connection between energy and health in, amongst other places, the bodies of coal miners, the air we breathe, and the toxic environments that surround us.^6^ Climate change, moreover, has revealed the threat to human health posed by our carbon addiction. When energy and health have been considered together, the focus has been on negative health impacts, rather than health as a product and driver of energy environments. And yet, as the history of New York’s Heat Code suggests, health and energy have been inextricably bound together though policy in the past. Not only did changing conceptualizations of health (as reflected in the Heat Code) shape the way energy was used; pressures in the mineral fuel network played a critical role in determining how comfort standards were codified in public health policy.

New York’s Heat Code acted as what Sarah Royston, Jan Selby, and Elizabeth Shove have described as an “invisible energy policy.”^7^ According to these authors, an “invisible energy policy” is a “non-energy policy,” which although not strictly emerging from the energy policy field, has unforeseen consequences and conflicting implications on energy demand and energy systems.^8^ Despite having a major impact on how New Yorkers consumed energy, the Heat Code was never formerly framed as part of New York’s “energy policy.”^9^ Instead, a range of institutions including the Department of Health, the City Council, the Fire Department, law courts, landlord associations, and tenant groups all came to shape New York’s energy environment by pushing for the creation of and adjustments to the Heat Code, increasing, and during the city’s periodic energy crises, reducing energy demand across the city. The Heat Code therefore demonstrates how policies emerging from other fields have played a central role in shaping energy environments.

The sixty-eight degree standard established in the Heat Code was never neutral.^10^ Rather it reflected changing attitudes towards the body, which impacted the way heat was managed. On its establishment, sixty-eight degrees was selected as a temperature that boosted efficiency, reflecting Taylorist conceptions of the body as a “human motor.” As the minimum was lowered to sixty-five degrees during World War II, purported optimum bodily comfort was abandoned for fuel conservation. The minimum was raised to sixty-eight degrees in 1956, as part of a wider agenda to eradicate dangerous secondary heating devices and centralize heat. These changing conceptualizations of what constituted a safe minimum reflected the shifting social demographics of the city, and the communities for which the Heat Code came to serve, which after World War II became increasingly Black and ethnic minorities. Attempts to develop a universal standard resulted in thermal discrimination, with certain communities suffering uncomfortable and often dangerous room temperatures. In affluent buildings, in stark contrast, overheated apartments were cooled with open windows leading to considerable energy waste.

By the 1960s, heat became part of a wider battle between communities and landlords over the urban fabric of New York. As fuel prices skyrocketed and the city confronted a fiscal crisis in the 1970s, the Heat Code became a way to enforce efficiency and cut the city’s ever-rising fuel bill. This reveals an alternative story from one of affluence and consumer abundance that came to define America’s post-1945 energy history.^11^ Contrary to the familiar stereotype of America’s “overheated” homes, many New Yorkers froze in apartments as landlords withheld heat, abandoned buildings, and tried to drive tenants out. During the 1970s, thermal inequity was endemic in the city, revealing how the lived experience of heat diverged between communities.

Sixty-eight degrees, therefore, served as a material point that bound health and energy together. Through it we can see the effects of one public health policy on the mineral fuel network of the city and how the constraints of that network brushed up against and informed health policy. The Heat Code, therefore, illustrates how policies that developed in other areas, such as health and housing, played a fundamental role in shaping energy demand, and how “invisible energy policy” facilitated our dependency on carbon and sharpened social inequalities.

## 1918: The Politics of Heat and the Establishment of Section 225

By 1900, New York was the second largest city in the world, with roughly 3,400,000 inhabitants. With bitterly cold winters, this growing city required ever more fuel to serve its expanding population. In the first decades of the twentieth century, the city’s heat was provided largely by coal—specifically, hard anthracite coal which was less polluting than its softer alternative, bituminous coal. New York State, however, had virtually no local coal sources. This made the city dependent on the coal fields in Pennsylvania to keep warm.^12^ Coal entered the city by rail and moved through wholesalers to retailers who sold coal in yards. This made for a highly volatile market with transport monopolies, labor disputes, and price manipulation being common problems. As a result, New York remained uniquely vulnerable to fuel shortages and disruption.

Between 1917 and 1926, World War I and a subsequent series of labor disputes in the mines and on the railroads accelerated the rate at which New Yorkers experienced coal famines. During the bitterly cold winter of 1917, the shortage of anthracite coal caused by rising wartime demand sent fuel prices soaring, leaving coal yards empty and causing coal riots across the city.^13^ New Yorkers, rich and poor alike, froze as they struggled to access the fuel needed to heat their homes. To mitigate the impact of the fuel shortage, the Federal Fuel Administration was established in 1917.^14^ This was one of the emergency administrations established during wartime as part of the Food and Fuel Control Act (otherwise known as the Lever Act). The Federal Fuel Administration oversaw the regulation of coal and oil during wartime, and established effective fuel conservation measures across the nation, including daylight-saving time. Each state had a Fuel Administration Board with local administrators, including the various boroughs of New York City. The New York branch of the Fuel Administration regulated the price of coal, implemented fuel conservation measures throughout the city, and ran a public education campaign on how to save fuel.^15^ Red priority tickets were also distributed (in partnership with the Fuel Committee of the Mayor’s Committee of Women on National Defense) to vulnerable families across the city to provide access to free coal and wood.^16^ [Insert WrightFig1 around here]

The 1917 coal famine marked a shift in the responsibilities of the Department of Health. Prior to the 1917, the Department of Health did not have a role in protecting New Yorkers from the cold. There were charitable organizations, including the Salvation Army and settlement houses who donated “charity coal.” Much of the fuel used by the poor, however, came from the streets through coal picking and wood scraps. Lack of fuel, therefore, was dealt with as a poverty issue solved through charity rather than viewed as a matter for public health. The Department of Health had taken on the responsibility of regulating semi-public spaces, including street cars and theatres, but this had yet to be extended to residential settings.^17^

Treating the cold as a public health menace nevertheless took on a new urgency in 1917, when heat became highly politicized, due to the unique way New Yorkers warmed their homes. At that time, New Yorkers lived in roughly three types of dwellings with different heating systems.^18^ Cold-water flats, such as tenement buildings, housed low-income tenants. These apartments were individually heated by a stove and tenants purchased fuel in addition to the rent. Heat was limited to one or two rooms. Due to limited storage, low-income tenants were forced to buy coal in smaller lots which were more expensive.^19^ Middle- and upper-class residents who lived in individual detached homes in Queens and Brooklyn also bought fuel to burn individually, although this tended to be in larger lots. Affluent and middle-class New Yorkers, in contrast, were increasingly living in centrally heated apartments, especially in high density areas with large apartment blocks such Manhattan and the Bronx. ^20^ These were fueled by a central furnace the basement, controlled by a janitor or building manager, who piped hot water and steam to apartment radiators throughout the building. The cost of heat was included in rent as a flat sum. Residents in centrally-heated apartments had no control over their heating systems or the temperature inside their apartments. It was this lack of control that made heat such a political issue, especially during the coal famine of 1917.

The lack of control over indoor temperatures led to an ongoing battle between landlords and tenants over what represented acceptable heating. One journalist summed up the argument:

Few landlords will agree with their tenants as to what is sufficient heat, and while humans require about the same amount of heat for health and efficiency, the average landlord regards his tenants’ requirements as those of a Sandwich Islander and the tenants construe the views of the landlord as those of an Eskimo.^21^

Prior to 1917, coal had little impact on the profit margins of landlords. When the price of coal rose rapidly, landlords struggled to access coal, declined to pay for it, or hoarded what became a precious commodity.^22^ Rent strikes were organized across the city as tenants refused to pay for apartments too cold to live in.^23^

The battle over heat lasted through the winter of 1917 and 1918, with landlords arguing they could not secure adequate coal for their tenants and accusing tenants of being unpatriotic in the call for excess heat.^24^ For their part, tenants had little legal protection from the cold. The law stated heat only had to be provided if it was stipulated in the lease agreement, and that was rare.^25^ Activist tenants across the city organized demands for more legal protections to heat. In February of 1918, a group of tenants met with Mayor John Hylan and the Board of Health to foreground the problem of unheated homes, recommending a range of protective measures.^26^ A bill was introduced by the socialist Assemblyman Samuel Orr, calling on landlords to maintain sixty-eight degrees in their buildings from October to April.^27^ Gradually, tenants won increasing rights to heat through several cases heard in New York’s Supreme Court, which ruled tenants did not need to pay full rent if no heat was supplied and granting tenants the right to vacate properties if they remained cold.^28^

It was the so-called Spanish Flu of 1918 that forced the Department of Health to take seriously the danger of cold apartments.^29^ The Spanish Flu led to a large expansion in the role of the Department of Health, as it implemented new public health measures across the city. The Heat Code was one of these measures, and its implementation coincided with the highest period of morbidity in the city. In October 1918, Section 225 was added to the Sanitary Code requiring a minimum temperature of sixty-eight degrees in places of residence of one or more persons “at all such times.”^30^

The creation of the ordinance led to a wave of heat complaints flooding into the Sanitary Board. In October 1918 there were 2405 complaints which were investigated by the Sanitary Bureau, 1605 being deemed valid and “abated by personal effort.”^31^ The continued failure of landlords to provide heat, nevertheless, shifted the Department of Health’s approach from “voluntary compliance” to pursuing criminal prosecution. In 1918, the Municipal Term Court upheld Section 225 and fined two landlords $100 (or a ten-day jail sentence) for failing to adhere to the code.^32^After this ruling, landlords would regularly be taken to court for their failure to provide heat. By December 1919, it was clear further clarification of the phrasing “at all such times” was needed. As tenants had interpreted this to mean round-the-clock heating, a new amendment specified that “unless otherwise provided by a contract or agreement, shall include the time between the hours of 6 A.M and 10 P.M.”^33^ This reduced the fuel burden on landlords, who only had to heat apartments to sixty-eight degrees during fixed hours.

As strikes and shortages continued between 1920 and 1926, the connection between coal and health became increasingly understood as a result of the labor disputes that punctuated the decade. Eventually, the reframing of coal as a basic necessity entered national politics. The most prominent advocate for this came from the New York Commissioner for Public Health, Royal Copeland, who classified “anthracite coal with air and water as essential to human life.”^34^ During the coal strike in 1920, Copeland had publicly threatened to seize coal for the purpose of keeping humans warm, using the police powers granted by the Department of Health if necessary.^35^ Maneuvering from the Department of Health into national politics, Copeland eventually ran for the U.S. Senate on a platform that promised to end the coal strikes menacing the health of New Yorkers.^36^ On entering the Senate in 1923, Copeland introduced bills to end the coal shortage by banning strikes and calling for the nationalization of the coal industry, or at least a permanent regulatory body to oversee it.^37^ In one public announcement he declared that “the nation cannot afford to face another coal shortage. Lower efficiency, spread of epidemics and disaster would follow.”^38^

## The Threat of “Overheating” and the Fuel Conservation Campaign

Although Section 225 established the cold as a public health menace, it also had a role in setting an upper limit to bodily comfort. This illustrates how Section 225 was created not just to protect tenants from the cold, but also to prevent “overheating.” One of the most influential studies to warn against the danger of overheating was the New York State Commission on Ventilation, headed by the Yale public health expert C.E.A. Winslow.^39^ Funded by the philanthropist Elizabeth Milbank Anderson, the commission provided a scientific study of the health effects of indoor climates in New York classrooms. Drawing on current trends in scientific management, the commission ran a series of experiments on the effects of indoor temperature on handwriting, memory recall, and physical exercise, to determine the optimum temperature for mental and physical work. Although the final report would not be published until 1923, its findings became well-known as early as 1917. It found sixty-eight degrees was the optimal temperature for human efficiency, with lowered levels of productivity when temperatures rose higher.^40^ The attempt to define the optimum thermal conditions for human efficiency reflected the growing effort to develop a scientifically codified “comfort zone,” a project that would be led by the American Society of Heating and Ventilating Engineers (ASHVE) after the war.

Other experts developed expansive theories about the danger of heat on human civilization, and in particular, the American nation. The geographer Ellsworth Huntington, also based at Yale, warned about the threat of overheating, tying this to a racial theory about the rise and decline of civilization. In his book *Climate and Civilization* (1915), Huntington extended the concern with overheating into a theory of racial superiority, with those living in warmer regions such as the American South described as being of lower vitality than those in cooler regions.^41^ Huntington believed the same effect was occurring in homes across the nation, where overheating was “sapping the energy and vitality” of Americans. In one study, he attributed high death rates in winter not to the cold but to overheating, suggesting that lower indoor temperatures would be critical in reducing morbidity.^42^ The racial overtones of Huntington’s theory was explicit and played into popular concerns circulating at the time about racial degeneration. For Huntington, white Americans who overheated their homes not only risked damaging their health, but also hindering the progress of the American nation.

During the 1917 fuel crisis, this rhetoric became a part of the discussion about fuel conservation, tying energy conservation into a racial discourse about progress and civilization. Public health experts, including Huntington and Winslow, became advocates for the health dangers of overheating.^43^ Huntington was even invited to Washington to advise the Federal Fuel Administration on indoor climates and write about the dangers of overheating for the fuel conservation campaign.^44^ In an article sponsored by the Federal Fuel Administration, Huntington went so far as to claim that “the shortage of coal will actually *improve our health*.”^45^ Returning to *Climate and Civilization*, Huntington recommended a minimum temperature of sixty-four degrees, stating that “if the coal situation should demand it, there is no reason why the temperature should not average as low as 60°. Indeed, even 56° would do less harm than 72°, which is a common temperature in many houses at present.”^46^

The call to avoid “overheating” echoed through the press and was popularized by notable figures, such as the future president Herbert Hoover.^47^ Hoover supported the sixty-eight degree standard and reiterated that not only would adhering to this temperature help save fuel but would also “better the health and physical resistance of the people.”^48^ The press, therefore, promoted the message that “health will be preserved, or even improved, with less heat.”^49^ Overheating also became highly moralized. As one journalist wrote:

Overheating, like overeating, is a vice and we should learn to recognize it. One cannot trust one’s own feelings, since when most Americans always know when they are too cold, they never know when they are too hot – they have absolutely no subjective upper limit in the matter of temperature.^50^

In July 1918, the head of the Federal Fuel Administration, Harry Garfield announced that households in New York and other large cities would only be allocated enough fuel “scientifically necessary” to heat the home to sixty-eight degrees through a rationing scheme. The system, the Fuel Administration explained, would be drastic but pledged that “no one will be deprived of coal actually needed for heating, but no one will be allowed fuel for waste or extravagance.” Garfield carefully differentiated between a “comfortable” temperature of sixty-eight degrees and the “thoughtless and wasteful” consumer who “will only have himself to thank if he has no fuel with which to heat his house.”^51^ Although household rationing would not be implemented, the Federal Fuel Administration sent a clear message that sixty-eight degrees was the optimum temperature that not only preserved health but also saved fuel. Sixty-eight degrees, therefore, represented an upper limit as much as a minimum.

## 1942-1956: World War II and the ‘Discomfort Zone’

Sixty-eight degrees was established as a wartime measure to preserve American efficiency, reflecting as much an upper limit to conserve fuel as a lower limit to preserve health. By 1942, as the outbreak of World War II stopped oil tankers from reaching New York, a new minimum emerged that abandoned the premise of optimum bodily comfort. By 1940, New York’s heat network had become increasingly reliant on oil, with 18% of residential dwellings using this as their primary heating fuel.^52^ The years of strikes and disruption in the coal mines and on the railways made oil a more reliable heating source, easier to transport and cleaner to use. New York’s housing stock was also changing, following a housing construction boom during the 1920s.^53^ The high cost of paying for fuel (on top of rent) meant that during the 1930s tenants had fled cold-water flats to centrally-heated apartments (a migration that operated seasonally as people moved back to cold-water flats in summer). Increasingly, to keep their tenants, landlords begun to recondition buildings, installing central heating systems.^54^ The cold-water flat was also under threat from a drive to modernize the city. Although in its nascent stages, the process of “urban redevelopment” (later termed “urban renewal”) led by Robert Moses meant that decrepit cold-water tenement buildings in so-called “slum” areas were knocked down and replaced by centrally-heated modernist apartment-blocks, a process that accelerated after the war with the passing of “Title I” of the 1949 U.S Housing Act. With 85% of residential dwelling having central heating by 1940, New Yorkers had become increasingly dependent on landlords for warmth.^55^

Wartime oil shortages, therefore, threatened to plunge New Yorkers into the cold once again. This time it was the Petroleum Administration for War (PAW) and the Office of Price Administration (OPA) that oversaw the distribution of oil. Like the Federal Fuel Administration, PAW had outposts in cities across the nation. Ernest Stebbins, the New York Health Commissioner, sat on the New York Committee of PAW. As fears over the oil shortage grew, an extensive campaign was launched to convert oil heaters back to coal. Rationing of fuel oil was also brought in by the OPA. The public was urged to voluntarily conserve fuel by shutting all doors and windows, and maintain heat only in rooms they were using, with all additional electricity devices turned off.^56^ As the shortage of oil became critical in 1942, the heating ordinance was lowered to sixty-five degrees.^57^ At first this applied only to oil fired apartment buildings, with sixty-eight degrees maintained in apartments heated by coal and gas. Sixty-five degrees, however, was soon extended to all buildings covered by the Heat Code.^58^

Once again the lower limit of sixty-five degrees was supported by medical practitioners who submitted their recommendations to PAW.^59^ Leverett D. Bristol, who wrote a report for PAW, justified sixty-five degrees not on the basis of the “comfort zone” but instead the “discomfort zone.” This was the point where health was not jeopardized but “some discomfort or suffering might be required.”^60^ European medical advice supported the lower minimum, with England and Germany referenced as places where indoor temperatures were kept much cooler, at around sixty degrees, with no serious repercussions to health.

E.A. Winslow continued to serve as a key authority on the heat question. During the war, however, he used updated scientific data from the Committee on the Hygiene of Housing, established by the American Public Health Association in 1938. This committee was founded to establish the “fundamental minima required for the promotion of physical, mental and social health, essential to low-rent as well as high-cost housing.”^61^ For the “normally vigorous person, normally clothed, and at rest,” Winslow advocated sixty-five degrees as the minimum temperature for the home. However, he created a separate minimum of seventy degrees for those of “subnormal vitality”: the infirm, the young, and the old. Given that most households were mixed, Winslow’s Committee recommended seventy degrees as optimal for the home.^62^ Winslow reiterated these standards when he spoke at an event as part of the War on Fuel Waste Week in August 1942, sponsored by ASHVE. He explained that people could, if necessary, remain healthy in temperatures between fifty-five to sixty degrees, if they also wore “heavy woolen underwear” and “ski suits”. He did recognize low temperatures would create additional problems for women, who liked temperatures to be five degrees warmer due to wearing lighter clothes. For vulnerable groups, Winslow added, “comfort may be provided in any atmosphere by the use of electrically heated blankets.”^63^

Sixty-five degrees, therefore, was not discussed as an optimum temperature but one that could provide adequate protection with necessary precautions. In fact, the secretary of ASHVE wrote to LaGuardia’s office in 1942 to complain that the medical advice that supported sixty-five degrees had been misinterpreted by the media and the OPA.^64^ Referencing, in particular, one *Life* article that made the “optimistic statement” that a “healthful temperature for active adults is sixty-five degrees,” he clarified that the medical consensus stressed that sixty-five degrees may only “be considered minimum temperature for emergency requirements of fuel oil rationing.”^65^

Those living in oil-heated apartments did feel temperatures drop, as landlords failed to provide adequate heat to meet the new minimum. When the heating ordinance was lowered in New York, heat complaints doubled.^66^ Letters from cold tenants poured into Mayor Fiorello LaGuardia’s office, protesting about the freezing conditions in apartments across the city. Some expressed a clear sense of injustice about how the conservation measures were being applied. One woman (who marched around the city with a thermometer) was dismayed by the high temperatures found in public buildings compared to her own apartment. She noted buildings such as City Hall were so hot, doors had been flung open and windows left open. She concluded that “the heating law, Mr Mayor, is a rich man law and a landlord’s law.” Pointing out the importance of “equality of sacrifice” for wartime morale, she urged the Mayor to modify the language in the Heat Code. She laid out a few suggestions, recommending that it should stipulate that a landlord should “not heat a building when the ‘street temperature’ is above 55°F or even 59°F, to prevent those in ‘POWER’ demanding heat even when the ‘street temperatures are as high as 65°.” As “street temperature” varied greatly in a city that expanded over thousands of acres, “enjoying a range of temperatures every day,” she proposed that an air raid siren should sound at 7 a.m. alerting landlords to days when they needed to turn their boilers on.^67^

Although the lower minimum alleviated some of the pressure on landlords, many worried that with access to only 66% of the normal oil quota, they lacked sufficient oil to heat apartments. One landlord of a building where a four-year old boy with asthma lived, questioned how he could keep all his tenants warm with only 430 gallons of fuel oil issued by the OPA. As his tenants were already careful to conserve energy, there was, he pointed out, an “irreducible minimum below which we cannot go.” Would the OPA, he asked, take responsibility if the child died as a result of the cold? ^68^ Other landlords were tired of tenants threatening to withhold rent and report them to the Department of Health for their failure to provide heat. The Associated Builders, which represented the owners of 1200 large apartments, demanded that the mayor be brave and tell the city’s tenantry that due to oil rationing property owners would be unable to provide the same temperature as in previous years.^69^ Taking a different approach, one New York resident urged the mayor to use the “police power” of the Department of Health to legally enforce landlords to convert their boilers from oil to coal to ensure the sixty-five degree minimum was maintained.^70^

## 1945-1956: Thermal Inequality and the Centralization of Heat

Despite being in the “discomfort zone,” sixty-five degrees remained the minimum until 1956. By this time a different social demographic was living in centrally-heated apartments across the city. In the post-1945 period, like other cities across the United States, New York experienced white flight as middle-class residents relocated to the suburbs, with low-income Black and ethnic minority communities moving into the city. Sixty-five degrees, therefore, became the new norm for low-income tenants, in accordance with wartime emergency fuel measures. There were appeals to the Board of Health to revert to the sixty-eight degree minimum (and even calls in 1950 to increase the code to seventy-two degrees).^71^ However, the Board of Health maintained there was no health hazard in having temperatures as low as sixty-five degrees.^72^ After an earlier appeal in 1947, the Health Commissioner Israel Weinstein even claimed that “the war made us a little more rugged” and so we are now able to withstand sixty-five degree temperatures comfortably.^73^

The fact that the sixty-five degree standard had been built into the heating code for over a decade after the war ended reveals the social inequities that came to structure the distribution of heat within New York. This was best phrased by one New York magistrate Charles F. Murphy, who as early as 1949, rebuked the City’s Board of Health for preserving the sixty-five degree minimum long after the fuel crisis had ended. In his words, “the 65 degree minimum was set by the Board of Health when they and the landlords found that a well-fed and well-clothed man could live at that temperature if he had to.” However, he went to say that “we are not all well-fed, and we are not all well-clothed. And even if we were, we could not lead a normal life in an apartment where the temperature is only 65 degrees.” He pointed out that those in New York City’s Board of Health lived in well-heated apartments: “I have never seen a tenant from these sections of the community come into court and complain of lack of heat and why should they? They get all the heat they wanted.”^74^

The complaint that the sixty-five degree standard discriminated against poorer tenants fell on deaf ears until 1954. The catalyst for this was the death of eight members of the Gonzales family, their two family friends, and their dog, recent immigrants from Puerto Rico, who died of carbon monoxide poisoning from a faulty water heater that they were using to heat their three-room apartment.^75^ The death of the Gonzales family drew attention to the dangerous gas and kerosene heaters used as primary and secondary heating implements, prone to poisoning tenants and starting fires. To prevent additional deaths, the Department of Health and the Fire Department called for the improved safety of gas devices and a ban on kerosene stoves, and initiated education campaigns to warn against the dangers of using these heating devices. To reduce accidents from secondary heating devices, the Department of Health placed pressure on landlords to maintain the minimum temperature of sixty-five degrees in flats.^76^As the issue intensified by 1956, the City Administration introduced a new law that cold-water flats in buildings of ten or more had to have central heating units installed with a central furnace. This was a critical component of Mayor’s Robert F. Wagner’s Multiple Dwelling Law, which sought to eradicate dangerous secondary heating devices by enforcing central heating in all apartments.

There were some who worried that the ban on gas and portable kerosene stoves would not help poorer tenants unless this was accompanied with legislation to install steam or gas heating as an alternative. The State Rent Administrator Charles Abrams, for one, predicted that low-income tenants would face rent rises as landlords confronted the costs of central heating. In his words, “death by freezing is a poor alternative to death by fire,” observing that kerosene stoves are the “sole means of heating for many people in the city.”^77^

Although the Department of Health claimed that the sixty-five degree standard had not caused any “illness or death” in November 1956, the Heat Code was amended back to sixty-eight degrees.^78^ Rather than being framed as an issue of bodily comfort, the reinstatement of the sixty-eight degree standard was intended to prevent dangerous living environments and property damage resulting from the rise of portable gas and kerosene heaters.

## 1956-1965: 24-Hour Heat and Air Pollution

The reinstatement of the sixty-eight degree standard in the Sanitary Code did not lead to increased heat for New Yorkers. On the contrary, landlords continued to ignore the Sanitary Code, leaving low-income tenants freezing. As property values collapsed in redlined areas, where neglected buildings violated numerous sections of the Sanitary Code, landlords abandoned buildings and forced tenants out. Withholding heat became a tool to drive people from their homes. In response, the 1960s witnessed a wave of urban activism as tenants and communities organized. Many went on rent strike and took over buildings, stubbornly living in freezing conditions rather than vacate apartments.^79^ Some fought back. Tenants on 110^th^ Street started a street fire to protest the lack of heat.^80^ Others formed co-ops to buy fuel and employed janitors to keep boilers running.^81^

The Department of Health recognized the severity of the urban heat issue, opening a twenty-four-hour complaint and enforcement hotline. Emergency Repair Squads were established in 1965 with the jurisdiction (after twenty-four hours of prior warning) to enter any property where heat or hot water wasn’t being provided to make repairs and to “supply coal, heating oil, gas or other fuel” to be billed to the landlord later.^82^ Temporary accommodation was also offered in hotels organized by the City.^83^ Few left their flats for these warmer environments, however, because of fear that empty homes would be robbed.^84^

Amidst the soaring number of heat complaints in the winter of 1965, Robert A. Low, a Democratic Councilman petitioned the Board of Health to amend the Heat Code so that landlords must provide round-the-clock heating.^85^ Low pointed out how “outdated” the current ordinance was; New York was “no longer the sleepy place it once was,” he noted. Television shows, movie houses, and theaters ran late into the night, and New Yorkers worked and studied at unconventional hours. Moreover, he noted, landlords were exploiting the situation by firing boilers as late as possible “but meeting heating standards by the time an inspector appears.”^86^ He asserted that “it is legal to cut off heat a few hours after most people have finished dinner and are relaxing, but it is morally wrong and the surest way to help people get and stay sick.”^87^ Despite complaints the amendment would increase fuel bills, it took effect on October 1 1965, stipulating that between the hours of six a.m. and ten p.m., a temperature of fifty-five degrees had to be maintained when temperatures dropped below forty degrees.^88^

Low’s twenty-four-hour amendment sat rather uncomfortably with his other crusade, improving New York’s air pollution.^89^ The years 1963 and 1966 had seen major air pollution events known as the New York City Smog, which drew attention to the negative health impacts of air pollution.^90^ A bill passed in 1966 placed controls on the burning of high-sulphur fuels and targeted incinerators and heating plants. The call for more heating, at a time when the city was trying to reduce pollution from heating plants, might appear counterintuitive. On closer inspection, however, the two agendas were aligned. In 1966, New York’s newly appointed Commissioner of Air Pollution called for greater centralization of New York’s heating plants. Looking to Staten Island, where the development of single-family homes was still in its early stage, he advocated the creation of a central steam plant that would heat multiple homes in the area. This was akin to district heating schemes which had been developed in Europe and in certain places in the United States since the late nineteenth-century. Centralization, he claimed, also had to be incorporated into the rebuilding plans of the city. The more that heat was centralized, the more it could be regulated to ensure it adhered to air pollution standards.^91^ The call to extend the heating ordinance to twenty-four hours, therefore, could be viewed as part of the shift towards the larger centralized systems in which low-sulphur oil could be better regulated under the city’s air pollution regulations.

## 1973-1977: Phase “0” and the Drive for Energy Conservation

In 1973, when President Richard Nixon urged the nation to turn their thermostats down to sixty-eight degrees, conserving energy would have been the last thing on the minds of New Yorkers, many of whom shivered in their apartments that winter. Instead, it raised concerns that “energy conservation” would provide an excuse, once again, for landlords to withhold heat from tenants.^92^ The oil embargo caused by the Arab-Israeli War hit New York hard, as fuel prices rose steeply, and landlords struggled to afford oil to heat their buildings. New York was in a perilous position, with 64% of households depending on heating oil for warmth, and ill-prepared for this crisis. ^93^ New York’s tax base had collapsed by the 1970s as middle-class citizens and industry left, unemployment sky-rocketed, and the city’s bill for social services ballooned out of control.^94^ With the city’s finances teetering on the brink, New York could not afford the rising fuel prices swelling its operating costs and threatening to send private landlords into insolvency. The fiscal situation was so severe that by 1975 New York was on the verge of bankruptcy, only to be saved by a federal loan that came with strict stipulations for reducing New York’s budget and dramatically cutting services.

As fuel prices rose in the winter of 1973, landlords called for the sixty-eight degree minimum in the Health Code to be lowered, stating if sixty-eight degrees was maintained, tenants would be left with no heat whatsoever by the end of the month.^95^ Their appeal was rejected by the Department of Health who stated their concerns over the “effects of a temperature drop when there is a possibility of a flu epidemic.”^96^ Landlords, however, became desperate as fuel prices climbed ever higher. In one week in January alone, fuel prices grew by 40%, from 24 cents a gallon to 34 cents.^97^ In desperation, a group of landlords stormed the midtown offices of the Saudi Arabian mission to the UN to protest high prices. In a separate action called “fuel preservation day,” landlords called for boilers to be turned off for a day of “servicing” to draw attention to the “plight of owners” unable to cope with the rising costs.^98^ Due to the severity of the crisis, Mayor Abraham Beame formulated a fuel-cost “pass along,” allowing a temporary rent increase in rent-stabilized apartments that reflected the rising costs of fuel.^99^

Enforcing the Heat Code to the letter became central to the city’s campaign to reduce its expenditure on heat. This was a tactic used by New York City Housing Authority (NYCHA), who as the city’s biggest landlord had to implement dramatic energy conservation measures across its projects to keep costs down. With 162,000 apartments across the city, 71% of them (162 projects in total) heated by fuel oil, NYCHA saw its oil bill almost double in 1974, to $38 million.^100^ The rising costs of oil had already pushed the Authority into a $17 million dollar deficit in 1972, forcing an unprecedented 7.5% rent rise across its apartments that year, to be repeated in 1974. By the end of the decade the rising cost of fuel and other services would push NYCHA to the brink of collapse.^101^

To reduce its fuel costs, NYCHA proposed Phase “0” in November 1973. At the heart of Phase “0” was an adjustment to the way managers and superintendents provided heat and hot water, ensuring they were “in strict accordance with the Board of Health requirements.” Prior to this crisis, the Authority, “out of its concern for the comfort and welfare of its tenants,” had been providing heat and hot water “well in excess of the requirements of the Board of Health and the New York City Housing Maintenance Code.”^102^ Phase “0” would ensure apartments were not heated a degree beyond sixty-eight degrees. The Authority implemented other measures as part of Phase “0,” including an extensive education campaign on energy conservation, as well as implementing efficiency measures across its buildings. Although the central program to monitor apartment temperatures through periodic spot-checks failed due to lack of co-operation from local mangers (making it unclear how many builders were adhering to Phase “0”) careful monitoring of fuel budgets revealed Phase “0” reduced consumption significantly. ^103^ The Authority consumed 16,616,074 fewer gallons of oil in 1974 than 1972, despite having an additional 1,764 apartments to heat.^104^ However, this success was not replicated when it came to gas and electricity, which increased 3%, year on year. This was attributed to, among other factors, tenants who turned to gas stoves as supplementary heating sources as room temperatures dropped.^105^ To assure tenants that the Authority was providing adequate heat as stipulated by law, NYCHA regularly published details of its Heating Policy in its *Housing Authority Journal*, reprinting the terms of the Heat Code.^106^ [Insert WrightFig2 around here]

In January 1977, when President Jimmy Carter urged all Americans to lower their thermostats to sixty-five degrees, New York’s Heat Code had not been amended to meet this new standard. Instead, landlords were reminded by the *New York Times* not to be moved by any “patriotic” or “less exalted motives” and turn the temperature down, else risk a hefty fine and even imprisonment.^107^ Carter’s appeal to lower the thermostat to sixty-five degrees, therefore, contravened New York City Law.

The winter of 1977 was cold. Landlords went into arrears and were no longer able to afford, or willing to pay for, heat. People across the city shivered in apartments that had been abandoned or fallen into receivership. With the city’s services cut to the bone, emergency repair squads were slow to check on buildings where no heat was reported. Getting heat into apartment buildings was “like fighting an epidemic,” as described Jane Benedict, Chairman of the Metropolitan Council on Housing.^108^

The situation was becoming increasingly dangerous. When temperatures reached one below zero Fahrenheit, two elderly residents died in a welfare hotel where no heat or blankets had been provided. The owner, already $53,000 in arrears, hadn’t fixed the boiler, which had failed to work properly since an $8,000 upgrade to meet air pollution standards.^109^ Sabotage of heating systems also became common, as landlords tried to push tenants out, and tenants who sought relocation in public housing made apartments too dangerous to inhabit.^110^ “It has been so cold here that even the rats have run off,” noted one tenant in a building where a hot water pipe had been cut with a hacksaw.^111^ Amidst this crisis, there was a call for sixty-five degrees to become the new minimum in the heating code so as to encourage landlords to “stretch their heating dollars.” As one reporter noted, “some heat when most people are awake is better than no heat at all.”^112^

Samuel Granville, the management director at NYCHA, recognized lowering the temperature would not necessarily lead to cost savings. “We’re fighting a battle now at 68° to keep tenants from turning on supplementary heating devices,” he pointed out.^113^ If lower standards were adopted, he predicted, tenants would switch on “electric heaters and stoves.” To meet this challenge, NYCHA ran an innovative trial to give tenants control of their indoor temperatures. The inability to control temperatures in apartments had led to a farcical situation whereby to meet the legal minimum, higher temperatures had to be provided across the board. Temperatures across buildings had never been even, and while some rooms were too cold, others (especially those above boiler rooms) were far too hot. To control room temperatures, tenants were opening windows or using secondary heating devices.

In 1975, NYCHA partnered with Honeywell, Inc, an engineering company and an early innovator of thermostatic temperature control, along with the Federal Energy Research and Development Administration, to pioneer a Radiator Valve Demonstration Study. This study sought to determine the potential of controlling heat in individual apartments through the installation of a thermostatic radiator NERV valve (a non-electric thermostat attached to radiators) in five NYCHA buildings. Tenants were promised the valve would not reduce the amount of heat to apartments but would meet the Health Code’s sixty-eight degree minimum, just with less waste, as the thermostat would regulate indoor temperatures to “the heat level you require.”^114^ Although only implemented at small scale, the study pointed to the difficulty of meeting energy conservation goals given the centralized heating systems within New York’s apartment buildings. Not only did the obligation to meet the minimum temperature of sixty-eight degrees lead to waste as tenants in warmer apartments opened their windows; it also pointed to another problem: people liked to live at different temperatures.

## Conclusion

The evolution of New York’s Heat Code provides an illuminating case study in how energy and health have been bound together in policy. The Department of Health had a pivotal role in shaping energy demand in New York City through the Heat Code. Framing the cold as a public health menace required the Department of Health to regulate the distribution of heat across the city, and the Heat Code became a key mechanism to establish an upper limit of comfort and reduce energy demand. This was never an equitable process but resulted in thermal inequality, leaving some in overheated apartments (with windows flung open) and others freezing in the cold.

The sixty eight-degree standard, therefore, bound bodies, material culture, the built environment and resource networks together. These arrangements changed over time, dependent on wider social, political, and cultural forces. By unpacking the social organization of heat over a fifty-year period, the ways that the regulation of the indoor environment shaped resource networks and commodity flows outside the home becomes clear. The sixty-eight degree minimum did not just protect New Yorkers from the cold but determined the amount of anthracite coal and later heating oil needed to keep the city’s many boilers running to a high enough temperature. Moreover, lowering the Heat Code by a few degrees, became a tool to relieve pressure on New York’s strained mineral fuel network at critical moments. Reframing the Heat Code as an “invisible energy policy,” as opposed to a public health policy, underscores the need to expand beyond the traditional subjects of energy history in order to understand the broader forces shaping the modern, carbon intensive world. Only by going back and uncovering these “invisible energy policies” does the degree to which energy dependency has been woven into the norms and regulations that have come to structure everyday life become apparent and prove inextricably intertwined with pressing issues of social justice.

## Footnotes

1 “Heat and the Home,” *Weekly Bulletin of the Department of Health, City of New York* 7, no. 46 (Nov 16, 1918), 360; *Sanitary Code of the Board of Heath of the Department of Health of the City of New York* (New York: Board of Health, 1920), 97-98.

2 The Heat and Hot Water Code is now part of the “Administrative Code of the City of New York” known as the “Housing Maintenance Code”, enforced by the New York City Housing Preservation and Development Department.

3 Environmental histories of New York have also overlooked the impact that the Heat Code (and the regulation of the indoor environment) had on the development of the city’s carbon network. See Ted Steinberg, *Gotham Unbound: The Ecological History of Greater New York* (New York: Simon & Shuster, 2014); David Stradling, *The Nature of New York: An Environmental History of the Empire State* (Ithaca: Cornell University Press, 2010).

4 Mayor Bill de Blasio, *One City: Built to Last: Transforming New York City Buildings for a Low-Carbon Future, Technical Working Report* (New York: Mayor’s Office of Sustainability, 2016), 10.

5 Larissa Nicholls and Yolande Strengers,“Heatwaves, Cooling and Young Children at Home: Integrating Energy and Health Objectives,” *Energy Research & Social Science* 39 (2018):1-9.

6 See Stephen Mosley, *The Chimney of the World: A History of Smoke Pollution in Victorian and Edwardian Manchester* (Cambridge: White Horse Press, 2001); James Rodger Fleming, *Toxic Airs: Body, Place, Planet in Historical Perspective* (Pittsburgh: University of Pittsburgh Press, 2014); Julie Sze, *Noxious New York: The Racial Politics of Urban Health and Environmental Justice* (Cambridge, Mass; MIT Press, 2007).

7 Sarah Royston, Jan Selby, and Elizabeth Shove, “Invisible Energy Policies: A New Agenda for Energy Demand Reduction,” *Energy Policy* 123 (2018): 127-135.

8 Ibid.

9 The regulation of the Heat Code by the Department of Health and then the Housing Preservation and Development Department shows how it has been framed as part of the city’s health and housing policy, rather than as part of the city’s formal energy policy.

10 There is a growing body of literature on the social construction of thermal comfort, and in particular, the diffusion of air conditioning. See Marsha Ackerman, *Cool Comfort: America’s Romance with Air Conditioning* (Washington: Smithsonian Institute, 2002); Elizabeth Shove, *Comfort, Cleanliness, and Convenience: The Social Organization of Normality* (Oxford: Berg, 2003); Jiat-Hwee Chang & Tim Winter, “Thermal Modernity and Architecture,” *Journal of Architecture* 20, no. 1 (2015), 92-121.

11 The narrative of consumer abundance and the rapid rise in energy consumption that framed the post-1945 period, has focused predominantly on the American middle-class and overlooked how uneven access to affordable energy was in American society during this period. See, for example, J.R McNeill and Peter Engelke, *The Great Acceleration: An Environmental History of the Anthropocene from 1944* (Cambridge Mass: Harvard University Press, 2014).

12 Mike Wallace and Edwin G Burrows, *Greater Gotham: A History of New York City from 1898 to 1919* (Oxford: Oxford University Press, 2018), 206.

13 See Robert M. Fogelson, *The Great Rent Wars: New York, 1917-1929* (New Haven: Yale University Press, 2013), 1-13.

14 Report of the Administrative Division, *Reports of The Bureau of State Organizations and of the Federal Fuel Administrators for the Various States and Districts, Part 1*, 1917-1919, ed., George Edwin Howes (Washington: Government Printing Office, 1920).

15 Ibid, 248-251.

16 *The Activities of the Mayor’s Committee of Women on National Defense, New York, 1918-1919* (1919), 24. “Gives Fuel to the Poor,” *New York Times*, Feb 17, 1918.

17 In 1915, “Section 55” Sanitary Code was amended to state that every “theatre, auditorium, assembly hall, factory, workroom, store, or office” shall “maintain proper degrees of temperature and humidity.” Arthur Cosby, *Code of Ordinances of The City of New York, including The Sanitary Code, The Building Code and Park Regulations* (Banks Law Publishing Company: New York, 1915), 378. In 1909, the Public Service Commission, encouraged by the Department of Health, established an ordinance regulating the temperature of the city’s street cars. This was added to New York’s Sanitary Code by the Department of Health in 1916 as part of “Section 304” regulating heating within street cars and other public vehicles. *Annual Report of the Department of Health of the City of New York*, 1916, 37.

18 For the most comprehensive history of New York housing see Richard Plunz, *A History of Housing in New York City* (Columbia University Press, 2016).

19 Winifred Gibbs, *The Minimum Cost of Living, A Study of Families of Limited Income in New York City* (New York: MacMillan, 1918), 86-88.

20 Plunz, *A History of Housing in New York City*, 78. Jared N. Day, *Urban Castles*: *Tenement Housing and Landlord Activism in New York City, 1890-1943* (New York: Columbia University Press, 1999), 94

21 “The Landlord, the Tenant, and the Heat Question,” *New York Times*, June 2, 1917, 60.

22 Day, *Urban Castles*, 96.

23 “The Landlord, the Tenant, and the Heat Question.”

24 “Bronx Landlords Plan Spring Drive on Non-Paying Tenants,” *New York Times*, Jan 28, 1918, 12.

25 Day, *Urban Castles*, 96.

26 Ibid.

27 Ibid, 98-99; Fogelson, *The Great Rent Wars*, 5-6.

28 “Not Liable for Cold Flats,” *New York Times*, August 4, 1918, 23.

29 John Duffy, *A History of Public Health in New York City, 1866-1966* (New York: Russell Sage Foundation, 1974), 287.

30 “Heat and the Home,” 360.

31 “Heat Complaints,” *Weekly Bulletin of the Department of Health, City of New York* 7, no. 48 (Nov 30, 1918), 375-376; “House Heat Regulations,” *Annual Report of the Department of Health of the City of New York, 1918*, 46.

32 “Heat in Apartments,” *Weekly Bulletin of the Department of Health, City of New York* 7, no. 52 (Dec 28, 1918), 408-409.

33 “Heating of Occupied Buildings Regulated,” *Weekly Bulletin of the Department of Health, City of New York* 4, no.1 (Jan 3, 1920), 1-2; “Regulation of Heating of Dwellings,” *Weekly Bulletin of the Department of Health, City of New York* 4, no. 17 (April 24, 1920), 132-133.

34 “Copeland for Ban on Miners Strikes,” *New York Times*, Dec 2, 1923, 6.

35 Duffy, *A History of Public Health in New York City*, 291; “Copeland Will Seize Coal if Necessary,” *New York Times*, Oct 17, 1920, 21.

36 “Copeland Replies Sharply to Binkerd,” *New York Times*, Feb 4, 1923, E2.

37 “Copeland Coal Plea Passes the Senate,” *New York Times*, Feb 1, 1926, 1.

38 “Copeland for Ban on Miners Strikes.”

39 New York State and Commission on Ventilation, *Ventilation Report of the New York State Commission on Ventilation* (New York: E.P. Dutton & Co., 1923).

40 *Ventilation Report*, 199.

41 Ellsworth Huntington, *Civilization and Climate* [1915] (New Haven: Yale University Press, 1924).

42 Ackermann, *Cool Comfort: America’s Romance with Air Conditioning* (Washington D.C., Smithsonian Books, 2010), 25-26.

43 “Advises Fuel Economy,” *The Washington Post*, Nov 30, 1917, 12.

44 Ellsworth Huntington, “Save Coal and Save Health,” *The American Review of Reviews* 58, no. 3 (1918), 287-291.

45 U.S. Fuel Administration, *Fuel Facts* (Washington DC: Government Printing Office, 1918).

46 Ibid.

47 A.M. Jungmann, “Save the Nation’s Energy-Fuel,” *Popular Science Monthly*, Jan 1919, 69-72.

48 “68 Degree Heat Limit for Home, Idea of Hoover,” *Chicago Daily Tribune*, Nov 28, 1917, 12.

49 “Houses Too Hot for Health,” *New York Times*, Nov 30, 1917, R9.

50 “Overheating,” *New York Tribune*, Jan 19, 1918, 10.

51 “Plans Completed for Coal Rationing,” *New York Times*, Jul 8, 1918, 18.

52 *Sixteenth Census of the United States: 1940, Housing, Volume Two, General Characteristics* (Washington: US Government Printing Office, 1943), 293

53 Plunz, *A History of Housing in New York City*, 122.

54 “Disconnected Notes, Heating Research”, “An Analysis of Heating Methods and Costs in Existing Minimum Cost Housing”, 14 Nov 1934, Box 55C7, Folder 08, New York City Housing Authority Collection, La Guardia and Wagner Archives. [From now on shortened to NYCHA Archive].

55 *Sixteenth Census of the United States*, 293.

56 “How to Keep Warm This Winter,” *New York Times*, Sep 27, 1942, 2.

57 “Minimum Heating Lowered in City,” *New York Times*, Oct 14, 1942, 27; “Rules for Heat in the City Explained,” *New York Times*, Nov 8, 1942, RE1.

58 Duffy, *A History of Public Health in New York City*, 376.

59 “Doctors Aid Fuel Rationing,” *Washington Post*, Oct 5, 1942, 17; “Parren Says 55 Degrees in Homes Would Not Hurt,” *New York Times*, Dec 3, 1942, 6.

60 Leverett Bristol, “Medical and Public Health Aspects of Heating Oil Rationing,” *ASHVE Journal, Heating, Piping and Air Conditioning* (Oct, 1942), 624.

61 American Public Health Association, *Basic Principles of Healthful Housing* (2nd Ed) (New York: American Public Health Association, 1941), 186.

62 *Basic Principles of Healthful Housing*, 187.

63 “‘Woolies’ Advised in Fuel Shortage,” *New York Times*, Aug 18, 1942, 24.

64 Letter from A.V. Hutchinson to F.J. Bloustein, Nov 4, 1942, Box 148, Folder 1, Office of the Mayor, Fiorello H. La Guardia, Box 146, La Guardia Archive, Municipal Archives, New York City [from now on shortened to La Guardia Archive]

65 “How to Heat Your House,” *Life* 13, no. 18 (Nov 2, 1942), 65-68.

66 “Complaints Rising on Lack of Heat,” *New York Times*, Dec 4, 1942, 15.

67 Letter from [M.F. Lanen?], Oct 31, 1942, Box. 3510, Folder 1, La Guardia Archive.

68 Letter assigned Dear Gentlemen, (no date), Box. 3510, Folder 1, La Guardia Archive.

69 Letter from the Associated Builders, Oct 28, 1942. Box. 3508, Folder 11, La Guardia Archive.

70 Letter from Douglas Scott Thropp to Mayor La Guardia, Nov 5, 1942, Box. 3508, Folder 11, La Guardia Archive.

71 “Res. 241,” Nov 21, 1950, The Council of the City of NY Collection, Box. 52714, La Guardia and Wagner Archives. [From now on shortened to The Council of the City of NY Collection].

72 John F. Mahoney to the Mayor, Jan 9, 1951. Box. 52714, The Council of the City of NY Collection.

73 “We’re More Rugged Now,” *New York Times*, Jan 15, 1947, 27.

74 “65 Heat in Homes Assailed by Court,” *New York Times*, Feb 12, 1949, 19.

75 “Gas Fumes Kill 10 in a 3 Room Flat,” *New York Times*, Nov 08, 1954, 1.

76 “Heating Rule Stressed,” *New York Times*, Jan 25, 1956, 16.

77 “Council Shelves Bill Limiting Oil Heaters,” *New York Times*, Oct 12, 1955, 1.

78 Law No. 722., Int, no. 660, Nov 27, 1956, *Proceedings of the Council of the City of New York*, 1956, 135. “Landlords Told to Increase Heat,” *New York Times*, Dec 1, 1956, 23; “Favour More Home Heat,” *New York Times*, Feb 16, 1956, 27.

79 “Bleak Day Spent by Rent Strikers,” *New York Times*, Dec 26, 1963.

80 “No Heat Protest Burns Up Street,” *New York Times*, Dec 29, 1966, 35.

81 “Tenant Prevents Building’s Decay,” *New York Times*, Dec 27,1967, 21.

82 “Law. No. 871,” Feb 16, 1965, *Proceedings of the Council of the City of New York* 1 (Jan 6 to June 29, 1965).

83 “City Offers Beds to Cold Tenants,” *New York Times*, Jan 29, 1966, 1.

84 “Heavy Snowfall Hits the City,” *New York Times*, Jan 30, 1966, 1.

85 The Sanitary Code was renamed the New York City Health Code in 1961. Res. No. 1275, March 2, 1965, *Proceedings of the Council of the City of New York*, 1. (Jan 6 to June 29, 1965).

86 Ibid.

87 “24-Hour Heating is Sought in City,” *New York Times*, Feb 19, 1965, 37.

88 “Landlords Heed New Heating Law,” *New York Times*, Nov 21, 1965, 64.

89 “Low to Seek Curb on Residual Oil Use,” *New York Times*, May 13, 1964, 22; “Pollution’s Nemesis,” *New York Times*, May 21, 1966, 21.

90 “City’s Dirty Air Called a Factor in Rising Deaths,” *New York Times*, June 23, 1965, 1; “Pollutants Tied to Flu Fatalities,” *New York Times*, June 23, 1965, 30.

91 “Multi-Building Heating Urged by Heller to Reduce Pollution,” *New York Times*, Dec 9, 1966, 1.

92 “68 degrees in City Dwellings Seen Assuring Fuel for January,” *New York Times*, Dec 20, 1973, 31.

93 *1970 Census: Housing, Volume I. Housing Characteristics for States, Cities, and Counties* (Washington: United States Government Printing Office), 34-189.

94 Kim Phillips-Fein, *Fear City: New York’s Fiscal Crisis and the Rise of Austerity Politics* (New York: Henry Holt & Co), 21-22.

95 “Landlords Seeking to Ease 68 Degrees Minimum to Save Fuel,” *New York Times*, Dec 7, 1973, 47.

96 Ibid.

97 “Landlords in City Report 40% Rise in Fuel Oil Price,” *New York Times*, Jan 17, 1974, 81.

98 “Landlords Stage Oil-Price Protest,” *New York City*, Oct 17, 1974.

99 “Excess’ Fuel Costs Would Be Shared,” *New York Times*, Dec 29, 1974, 215.

100 Office of the State Controller Division of Audits and Accounts, “Audit Report on Fuel Oil Deliveries and Usage at NYCHA Projects, Report No. NYC-94-75. Box 00884, Folder 5”, NYCHA Archive.

101 See Nicolas Dagen Bloom, *Public Housing that Worked: New York in the Twentieth Century* (Philadelphia: University of Pennsylvania Press, 2008).

102 Sidney Shackman, Memo to all Managers and Superintendents, “Fuel Conservation,” Nov 21, 1973, Box 00884, Folder 5, NYCHA Archive.

103 “Audit Report on Fuel Oil Deliveries and Usage at NYCHA Projects.”

104 John Simon, Memo, “Energy Conservation Program,” Dec 1975, Box 00884, Folder 5, NYCHA Archive.

105 Ibid.

106 “Cold Weather Ahead!,” *The Housing Authority Journal* (New York City Housing Authority) 4, no.6, February-March 1975; See “Here is Our Heating Policy,” repeated in *The Housing Authority Journal*, October-November, 1976, January-February, 1977; October-November, 1977. Box 0099D2, NYCHA Archive.

107 “Saving Heat Could Mean the Cooler,” *New York Times*, Jan 26, 1977, 27.

108 “Apartments’ Ordeal,” *New York Times*, Feb 06, 1977, R1.

109 “Hotel Where 2 Died Gets 300 Blankets,” *New York Times*, Jan 21, 1977, 13.

110 “Sabotage Cited in Some New York Heat Failures,” *New York Times*, Feb 01, 1977, 16.

111 Ibid.

112 “Cold Comfort on Heat,” *New York Times*, Jan 28, 1977, 17.

113 “Saving Heat Could Mean the Cooler.”

114 “Memo: The NERV Centre: A Winning Formula for Heat Control,” Feb 15, 1977, Box 0092E4, Folder 21, NYCHA Archive.

